# Spatio-temporal evolution characteristics analysis and optimization prediction of urban green infrastructure: a case study of Beijing, China

**DOI:** 10.1038/s41598-022-14613-z

**Published:** 2022-06-23

**Authors:** Yin Ma, Xinqi Zheng, Menglan Liu, Dongya Liu, Gang Ai, Xueye Chen

**Affiliations:** 1 School of Information Engineering, China University of Geosciences, Beijing, 100083 China; 2 Technology Innovation Center for Territory Spatial Big-Data, MNR of China, Beijing, 100036 China; 3 Beijing Fangshan Observation and Research Station of Comprehensive Exploration Technology, Ministry of Natural Resources of People’s Republic of China, Beijing, 102400 China; 4 Shenzhen Research Center of Digital City Engineering, Shenzhen, 518034 China; 5 Key Laboratory of Urban Land Resources Monitoring and Simulation, Ministry of Natural Resources, Shenzhen, 518034 China

**Keywords:** Urban ecology, Urban ecology, Sustainability, Environmental impact

## Abstract

The reasonable layout of green infrastructure is conducive to the low-carbon, livable and high-quality sustainable development of cities. The framework of spatio-temporal evolution characteristics and prediction analysis of Urban Green Infrastructure (UGI) was constructed by integrating morphological spatial pattern analysis (MSPA) and CA-Markov in the study. We analyzed the spatio-temporal evolution characteristics of UGI in Beijing from 1990 to 2019, predicted its future change trend in 2030, and put forward the optimization scheme for the ecological network of UGI. The area change of UGI presented a "V" shape from 1990 to 2019 in Beijing, and the turning point was around 2009. Its spatial distribution revealed a significant heterogeneity. The comprehensive change rate index showed a "rising and then falling" trend from 1990 to 2019. Core with an area of over 1000 km^2^ had inclined "C" shape, connecting the north, west and south of the study area. Among the three prediction scenarios for 2030, the area of UGI under the ecological conservation priority scenario is the largest, accounting for 86.35% of the total area. The area of UGI under the economic development priority scenario is the smallest, accounting for 76.85%. The optimization of zoning and road network are effective measures to improve the connectivity of UGI in Beijing. This study is beneficial to extend the research ideas of UGI and promote sustainable urban development.

## Introduction

In recent years, more and more people are pursuing high-quality urban life in order to get more happiness^1–3^. At the same time, people are not only concerned about urban environmental issues, such as urban heat island^4^, air pollution^5, 6^and flood disasters^7^, but also gradually pay attention to the theme of low-carbon, health, livability and high-quality development of cities^3, 8^. Therefore, the construction of the new theme city in the new era is an urgent task for the current research on urban issues. However, UGI can solve many problems in cities and has a prominent role and importance in sustainable urban development. The habitat around UGI has increased vegetation cover to improve urban ecosystem services^2, 9^. UGI can reduce the impact of climate change on cities by increasing carbon sequestration^10^. Some studies have shown that UGI is beneficial in reducing the incidence of diseases and crime rates^11^. In addition, UGI allows local detail planning and potential integrated planning among other urban settlements^12^.

The development of green infrastructure (GI) was heavily influenced by greenways and ecological networks, the concept of which was gradually defined in the 1990s^13–15^. With the attention and research of experts in various fields, there were different views on the definition of GI. The emphasis of the definition differs depending on the content of the study. Among them, the definition of GI which is ‘‘an interconnected network of natural areas and other open spaces that protects the values and functions of natural ecosystems, sustains clean air and water, and provides a wide range of benefits to people and wildlife’’ was widely used by ecologist and urban researcher^13, 16^. Based on existing research, UGI refers to the interrelated and organic unified green space network composed of natural and semi-natural areas in cities, including forest lands, cultivated lands, grasslands, watersheds, wetlands, parks and urban greening in this study^17^. The network can provide a source for animal migration and ecological processes, enhance ecosystem health and resilience, maintain bio-diversity and ecosystem balance, and provide benefits for human welfare^14, 15^. Therefore, it is of great practical significance to construct research framework of UGI, explore its spatio-temporal evolution characteristics, optimize ecological network, and enhance its stability and connectivity for high-quality urban development^18–20^.

After nearly 30 years of development, the identification of UGI has made great progress. Various methods have been developed and applied from the perspective of landscape science and urban ecology^17, 21^. Some scholars identified UGI through ecosystem services and source sink theory^22^. Some scholars have also explored the shape and distribution of UGI using the MSPA identification method based on mathematical morphology^18^. With the development of data diversification, remote sensing information extraction methods were also used for the identification of UGI^23^. Among, the common identification method was MSPA, a mathematical morphology algorithm developed by Vogt^18–20^. The method mapped individual pixel categories to different classes, such as cores, bridges, loops, branches, perforations, and edges^24^. On the basis of UGI’s identification, Fragstats and ArcGIS plug-in tools were used to calculate the patch density of each category of UGI^25^ and the minimum cost distance to describe the fragmentation and connectivity of UGI^26, 27^. Based on the above identification methods and tools, scholars have explored the evolutionary characteristics of UGI to diagnose its development and distribution problems for the optimization of UGI. In detail, some scholars have reflected the change of UGI by analyzing the evolution characteristics of urban forest^28, 29^. Other scholars have used the image data in 2003 and 2018 to identify total area changes of UGI and impacts on habitat connectivity in Stockholm, Sweden, and the research results provided data and ideas for planning optimization of UGI^23^. Studies have either analyzed the evolution of UGI as represented by the evolution of urban forests or have focused more on the overall spatio-temporal variation of the types of UGI. But fewer studies have analyzed major types, rates of change, and barycenter migration in time series.

UGI was attracted extensive attention from researchers in various disciplines. Urban ecology researchers have used expert opinion method^30^, ecosystem services assessment method^31^ and natural capital model method^4^ to study the quantitative relationship between UGI and urban ecosystem. Atmospheric experts have paid more attention to the relationship between UGI and air quality. Some scholars have used the integrated dispersed-deposition modelling and the weather research and forecasting model-community air quality modeling system to explore the linear or more complex relationships between UGI and air quality^32, 33^. Soil experts have believed that soil, water, climate and vegetation were a unified organic whole, and paid attention to the relationship between UGI and soil moisture through field monitoring methods^34^. Forest specialists were more concerned about how to plan and design vegetation to make the most out of UGI^28, 29^. In addition, some scholars have explored the assessment methods of UGI^35, 36^, the relationship between UGI and poor areas^37^, the role of UGI in urban flood management^7^ and promoting the construction of green roofs and green walls^38^. The prediction of UGI is also of particular interest. Fewer scholars have conducted related studies. Lin et al. used the CA–Markov model to predict the future spatial pattern of green infrastructure under three different scenarios, and the results of the study can provide basis and reference for the optimization of green space in the study area^39^. Diana et al. and Charlotte et al. also used CA–Markov model to achieve the prediction of UGI, and to quantitatively explore the relationship between green infrastructure and urban planning^40^. However, there is still much potential research space to promote high-quality sustainable urban development through development pattern trend prediction of UGI.

In order to fill the gap in the detailed study of UGI and its future development trend prediction, this study integrated the ideas of mathematical morphology and prediction to build a research framework. Specifically, we constructed a framework which is based on MSPA and CA-Markov to analyze the spatio-temporal evolution characteristics and predictions of UGI, and proposed two indicators to quantify the evolution characteristics of UGI. Moreover, the spatio-temporal evolution of UGI in Beijing from 1990 to 2019 was explored, the development trend in 2030 was predicted under three different scenarios, and the distribution characteristics, connectivity and barycenter migration trends of the core were analyzed, so as to better optimize urban ecological network and promote urban livability.

## Results

### Spatio-temporal evolution of UGI from 1990 to 2019

**Figure 1:**
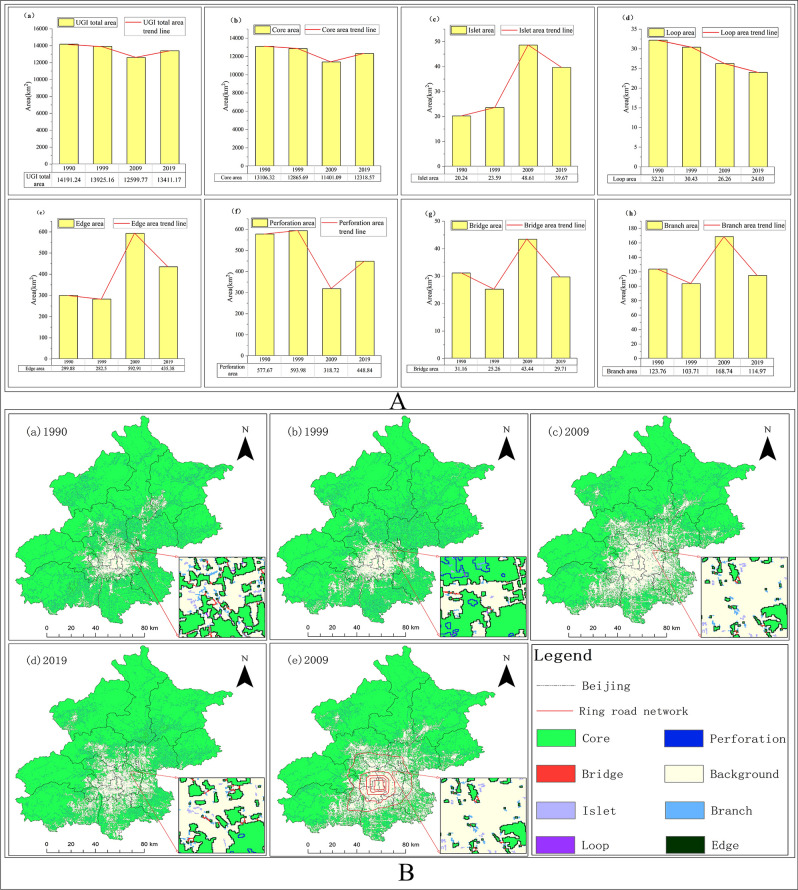
Spatio-temporal evolution of UGI in Beijing from 1990 to 2019. (**A**) Area variation of categories of UGI in Beijing from 1990 to 2019. (**B**) Distribution of UGI in Beijing from 1990 to 2019 and overlay map of UGI and sixth ring road network in 2009. The figure was drawn with ArcGIS sofware version 10.4. (https://www.esri.com/en-us/arcgis/products/arcgis-desktop/overview).

According to statistics, the area of UGI accounted for more than 76% of the total area of the study area from 1990 to 2019. The area of core was the largest of the seven categories, accounting for more than 90% of the total area of UGI, while island, bridge and loop were smaller, accounting for only about 0.2% of the total area. As shown in Fig. 1, the area of total and core showed a "V" shaped change trend from 1990 to 2019, with the turning point around 2009(Fig. 1A(a),(b)). The area of the island presented an inverted "V" shaped change trend, which was opposite to the change trend of the core. The turning point of its change also appeared around 2009 (Fig. 1A(c)). Moreover, the area of loop decreased year by year (Fig. 1A(d)). We found that there was no clear regularity in the trend of area changes of bridge, branch, edge and perforation (Fig. 1A(e)–(h)).From the perspective of network ecology of UGI and urban ecological integrity, there was a correlation between categories with edge and connectivity effects and those with ecological source and ecological island jump effects, and the trends of area changes were opposite. Just as in 2009, the area of core was the smallest during the study period. On the other hand, the branch and bridge had the largest area to ensure the connectivity and integrity of UGI. From the perspective of change rate index, as shown in Table 1, the comprehensive change rate index showed a "rising and then falling" trend, with 0.85% for 1990–1999, 1.00% for 1999–2009, and 0.90% for 2009–2019. From 1999 to 2009, the single change rate index was larger, in which the change rate of the core was -1.14%, and the area of core decreased significantly during this period. Moreover, the change rates of island, edge and bridge were 10.61%, 10.99% and 7.20% respectively, and their area had a tendency to increase.

**Table 1:** Single and comprehensive change rate index of UGI in Beijing from 1990 to 2019.

Date	Single change rate index							Comprehensive change rate index (%)
	Core (%)	Islet (%)	Edge (%)	Bridge (%)	Loop (%)	Perforation (%)	Branch (%)	
1990–1999	−0.20	1.84	−0.64	−2.10	0.61	0.31	−1.80	0.85
1999–2009	−1.14	10.61	10.99	7.20	−1.37	−4.63	6.27	1.00
2009–2019	0.80	−1.84	−2.66	−3.16	−0.85	4.08	−3.19	0.90

The spatial distribution of UGI in Beijing from 1990 to 2019 was shown in Fig. 1B(a)–(d). The ecological network and background areas of UGI showed obvious "ring" distribution characteristics in Beijing. The distribution characteristics are consistent with the circular radial greenway system layout of "three ring guidance, multi corridor connection and multi-layer expansion" proposed by relevant departments. The outer ring was ecological network of UGI, which is distributed around the study area. The inner ring, as the background area, was mainly distributed in the main urban area of Beijing. In this region, extensive construction land and a high-density road network resulted in a sparse distribution of UGI. In the time series of the study, the background area showed a trend of "expansion followed by contraction" centered on the main urban area. In detail, there was a trend of annual expansion from 1990 to 2009, and a clear trend of contraction from 2009 to 2019, especially in the eastern part of Chaoyang and the northern part of Haidian. The characteristic is mainly influenced by the policies enacted between 2011 and 2014 regarding the preservation and expansion of scenic garden construction such as historical and cultural scenic areas. Thus, it can be seen that the increase of green area in the parks is beneficial to enhance the connectivity of UGI within the city. On the contrary, the ecological network of UGI showed a trend of "contraction before expansion". From the perspective of Beijing's unique transportation network (Fig. 1 B(e)), the ecological network of UGI in the sixth ring road was relatively fragile, while the ecological network in the suburbs was relatively stable. The finding is consistent with the location of the second green barrier in Beijing as proposed in the greening policy promulgated in 2003. The construction of the green barrier has an important influence on the overall distribution characteristics of UGI in Beijing.

### Analysis of major cores from 1990 to 2019

**Figure 2:**
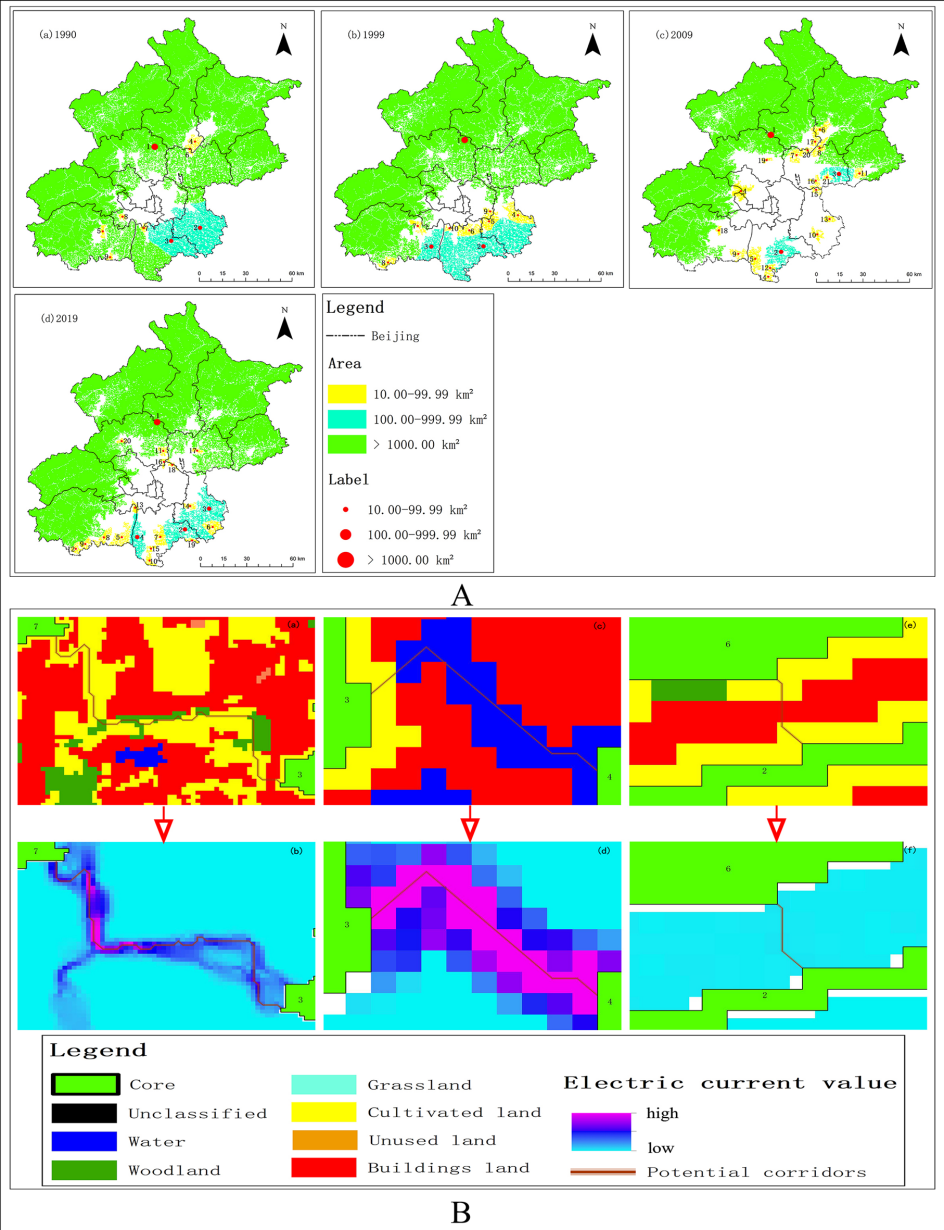
(**A**) Distribution of major cores of UGI in Beijing from 1990 to 2019. The figure was drawn with ArcGIS sofware version 10.4. (https://www.esri.com/en-us/arcgis/products/arcgis-desktop/overview). (**B**) Potential corridors and pinch points between major cores. (**a**,**c,e**) represent the potential corridors between major cores and the land cover of the potential corridor crossing. (**b,d,f**) represent the current values between the major cores, and the large current value indicates a significant pinch point. On the contrary, it indicates that the pinch point is not obvious.

From 1990 to 2019, the area of cores greater than 10 km^2^ accounted for more than 90% of the total area, and the ecological source area of 10 km^2^ can also provide a relatively stable ecological environment for organisms. From the perspective of proportion and ecological function, the cores with an area of more than 10 km^2^ were studied as major cores (Fig. 2A). On the whole, the major cores were mainly located in the suburbs surrounding the study area, while there was no major cores in the city center. Core (No. 1) with an area of over 1000 km^2^ had inclined "C" shape, connecting the north, west and south of the study area. The major core played an important role in Beijing because of its wide coverage and connectivity. The cores with an area of 100–1000 km^2^ were mainly distributed in the southeast of the study area, including the southern of Tongzhou, Daxing and the eastern of Fangshan, with smaller numbers. The cores with an area of 10–100 km^2^ were scattered and independent. Their number was more than the number of cores with larger area. In the study time series, the area, number and distribution of the major cores changed steadily from 1990 to 1999. However, the network of the major cores was affected in 2009. Specifically, the main manifestations were the decrease in total area, the significant increase in number, the large degree of ecological fragmentation, and the weak connectivity, especially in the central urban area, Tongzhou and Daxing. By 2019, the overall situation of the major cores were improved, which was shown by an increase in total area and the enhancement of connectivity. The ecological restoration effect of Daxing and Tongzhou was evident. However, the degree of ecological fragmentation was still large.

Based on the analysis of the potential corridors and pinch points between the major cores, we found that they were mainly distributed in four types of landscapes: cultivated land, water area, construction land and forest land. There were three main scenarios: (1) the potential ecological corridor between core (NO. 3) and core (NO. 7) was dominated by cultivated land and supplemented by forest land (Fig. 2B(a)). The ecological corridors were more likely to pass through woodland and cultivated land if they do so. The current value was large, and there were multiple paths connecting the two major cores with distinct pinch points and strong connectivity (Fig. 2B(b)). (2) The potential ecological corridor between core (NO.3) and core (NO.4) was dominated by water area (Fig. 2B(c)). Although the current path was single, the current value reached the maximum (Fig. 2B(d)). The water area can enhance the connectivity of cores and consolidate the ecological network of UGI. (3) In addition, potential ecological corridors existed between the cores of the construction land (Fig. 2B(e)). This potential ecological corridors had greater obstacles, smaller current value between the cores, weaker connectivity, and no obvious pinch points (Fig. 2B(f)).

### Prediction analysis of UGI in 2030

In order to verify the feasibility of the CA–Markov model in IDRISI 17.0 to predict the land use and cover data of Beijing in 2030, the study used the model to predict the land use and cover data of Beijing in 2009 and 2019. Specifically, the actual data of 1990 and 1999 were used to predict the data of 2009, and the actual data of 1999 and 2009 were used to forecast the data of 2019. Then, the consistency between the predicted and actual data for 2009 and 2019 was verified, and the kappa coefficients of the prediction accuracy were 0.8277 and 0.7877 respectively. The prediction accuracy of the CA–Markov model met the requirements of further research. Three development scenarios were proposed based on Beijing's development vision and national policies. Under the ecological conservation priority scenario, the transfer of elements within the ecological conservation red line was restricted. Elements included mainly forest land, water area and cultivated land. The effect of altitude and slope should also be considered. Under the economic development priority scenario, the transfer of construction land was restricted. In addition, the probability of transferring out of unused land should be increased. Again, the effects of elevation and slope were to be considered. The natural development scenario did not consider the influence of policies and the constraints of conversion between land types. After generating a scenario-specific land use suitability atlas, the CA–Markov model was applied to simulate each of the three scenarios. The land use prediction results were converted into elemental data of UGI by MSPA.

**Figure 3:**
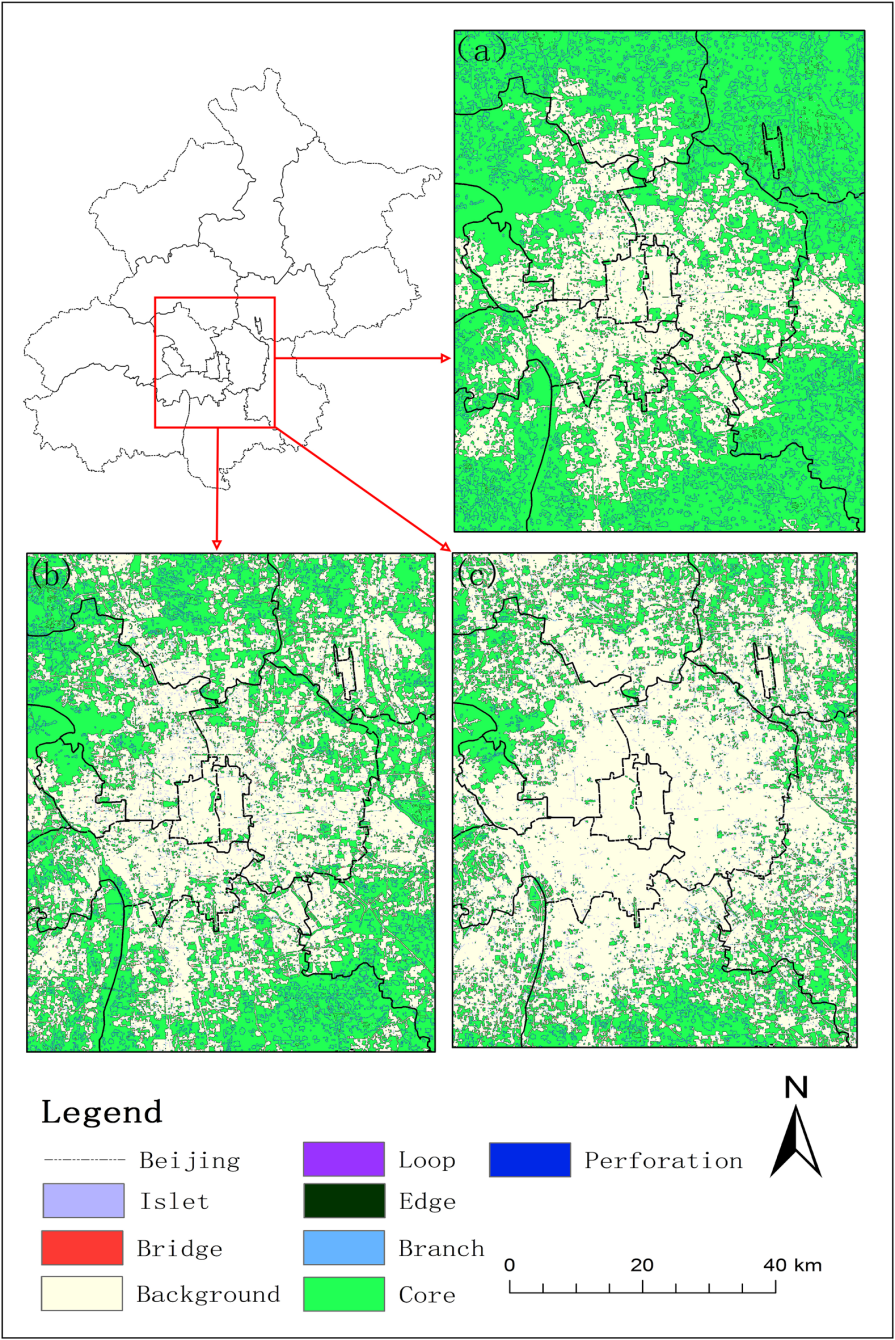
Distribution of UGI in 2030 in Beijing (**a**) ecological conservation priority scenario; (**b**) natural development scenario; (**c**) economic development priority scenario. The figure was drawn with ArcGIS sofware version 10.4. (https://www.esri.com/en-us/arcgis/products/arcgis-desktop/overview).

**Figure 4:**
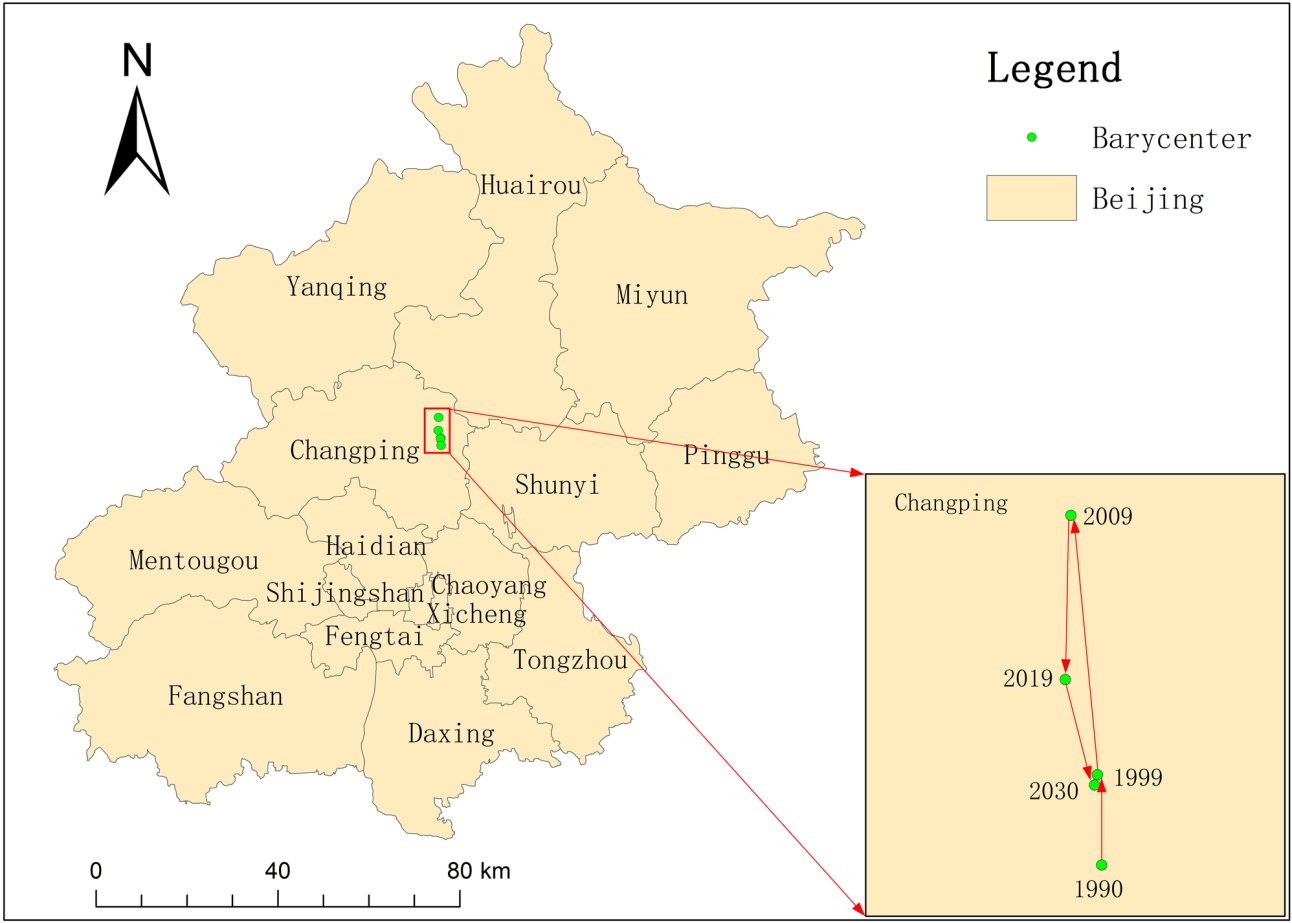
Barycenter migration of cores in Beijing from 1990 to 2030. The figure was drawn with ArcGIS sofware version 10.4. (https://www.esri.com/en-us/arcgis/products/arcgis-desktop/overview).

As shown in Table 2, the total area of UGI and the change rate of each type under the three development scenarios are significantly different. Under the ecological conservation priority scenario, the total area of UGI in Beijing in 2030 is 14,155.50 km^2^, which is 744.3 km^2^ more than that in 2019, accounting for 86.35% of the total area of the study area. The percentage of the area of the core reaches 95.45% of the total area of the UGI, while the total percentage of the other categories is only 4.55%. Under the natural development scenario, the total area of the UGI is 13,426.03 km^2^, accounting for 81.89% of the total area. It is comparable to the area in 2019, but is 729.47 km^2^ less than the area under the ecological conservation priority scenario. The total area of the UGI under the economic development priority scenario is 12,598.46 km^2^, accounting for 76.85% of the total area of the Beijing. In comparison with 2019, the area under this scenario is reduced by 812.71 km^2^. In terms of the change rate, the economic development priority scenario have the largest comprehensive change rate index of 0.82%. The ecological conservation priority scenario is the next largest, at 0.49%. The smallest combined rate of change index is 0.17% for the natural development scenario. From the above analysis, it is clear that the policy and development constraints have a greater role in regulating the change of UGI. From the development plan of Beijing, economic development and ecological optimization need to be carried out simultaneously in the next ten years. In addition, the spatial distribution of UGI in urban center of Beijing is widely different under the three scenarios, as shown in Fig. 3. Among them, the background area of the urban center is the largest in scope under the economic development priority scenario in comparison with the other two development scenarios. The connectivity of UGI is severely affected, and there is almost no UGI in the eastern and western urban areas. Under the ecological conservation priority scenario, the distribution of UGI in the central city is significantly broader and more connected. In consideration of the fact that the core accounts for a large proportion of the total area of UGI, the migration of its barycenter can reflect the overall change of UGI. The core in 2030 under the ecological conservation priority scenario and the core in the previous period are selected for the barycenter migration study. As shown in Fig. 4, from 1990 to 2030, the barycenter of cores is in Changping, showing a trend of "from south to north, then from north to south". The turning point of barycenter migration direction is around 2009, which further indicated that ecological network of UGI is gradually improving towards the urban center after 2009. In other words, in the future development, the policy and system of eco-environmental protection need to be given high priority in order to ensure the spatial distribution pattern and area of UGI.

**Table 2:** The area and rate of change of UGI in Beijing in 2030.

Multi-Scenarios	Type	Core	Islet	Edge	Bridge	Loop	Perforation	Branch	Total	UGI percentage of total area (%)	Comprehensive change rate index (2019–2030) (%)
Ecological conservation priority scenario	Area (km^2^)	13,511.48	13.42	209.78	5.69	20.51	346.15	48.47	14,155.50	86.35	0.49
	Percentage	95.45%	0.10%	1.48%	0.04%	0.14%	2.45%	0.34%	100%		
Natural development scenario	Area (km^2^)	12,340.17	38.33	432.00	28.87	24.56	448.96	113.14	13,426.03	81.89	0.17
	Percentage	90.49%	0.39%	4.71%	1.34%	0.21%	2.53%	1.34%	100%		
Economic development priority scenario	Area (km^2^)	11,399.86	48.70	592.93	43.41	26.27	318.65	168.64	12,598.46	76.85	0.82
	Percentage	91.91%	0.29%	3.22%	0.21%	0.18%	3.34%	0.84%	100%

## Discussion

As for the research framework of UGI, several scholars have studied it from the perspectives of planning and management^1^, renovation and restoration^25^, identification and rendering^41^. We explored the spatio-temporal evolution of UGI from a historical-present-future long time series perspective. Our goal was to provide ideas for analysis and optimization of UGI at the urban scale, and also to support sustainable development of low-carbon, healthy, livable and high-quality cities. In order to ensure the consistency of the before-and-after data, and make the research more meaningful, we chose 30 m resolution images of the Landsat series. However, there were limitations of the 30 m resolution data for the study. For example, the data cannot better represent the spatio-temporal characteristics of UGI within the street and community scales. In other words, it is unable to characterize the fine-grained features of UGI. In the future, we will use higher resolution data to conduct studies of UGI at the street scale or community scale in Beijing. Besides, it was a new attempt to integrate CA–Markov model into the study of spatio-temporal evolutionary characteristics of UGI. With the support of the prediction model, the future development trend and general situation of the study area can be well known in advance, which provided data support for policy formulation and optimization scheme design. Although our study has conducted a multi-scenario prediction analysis, there are some limitations in terms of the constraints perspective. In detail, the evolution of UGI is influenced by multiple factors. In addition to the constraints currently considered in the study, it may also be affected by natural disasters and unexpected situations.

Beijing is surrounded by mountains on three sides in the west, north and northeast, and forest land and cultivated land are widely distributed. This is the main reason that the area of UGI in Beijing accounted for more than 76% of the total area of the study area. The unique topography of the study area with high northwest and low southeast also affected the distribution and connectivity of the major cores in UGI^42, 43^. The cores in high terrain areas were widely distributed and highly connected, while the cores in low terrain areas were sparsely distributed and weakly connected. We found that the area and distribution centers of UGI changed significantly in 2009. In the past 20 years from 1990 to 2009, the country's development centered on economic construction, and a large influx of people into cities, which accelerated the rate of urban expansion. In order to meet the economic development and the housing for a large population, the area of construction land had increased rapidly, and it had been achieved mainly by expropriating cultivated land around the former urban area^42, 44^. Insufficient attention has been paid to the balance of environment, resulting in a reduction of urban green space. Urban expansion mainly occurred in the main urban area centered on the Dongcheng and Xicheng districts, while the green base was widely distributed in the northwest of the entire study area, so the barycenter of UGI shifted northward from 1990 to 2009, as shown in Fig. 4. With the inclusion of ecological civilization construction in the “five-sphere integrated plan” national development strategy in 2012, we have paid more attention to ecological construction from the national level. In addition, the implementation of afforestation and demolition of illegal buildings has increased the area of the green space in Beijing, which has increased the area of UGI from 2009 to 2019, as shown in Fig. 1A(a). This also caused the distribution of barycenter of UGI to shift from north to south, as shown in Fig. 4. The prediction study found that the area of green space increased under the natural development scenario and ecological conservation priority scenario, while it decreased under the economic development scenario. Liu et al. and Li et al. also came up with similar findings^45, 46^. In conclusion, some policies are needed to guarantee the area and the connectivity of ecological network of UGI.

**Figure 5:**
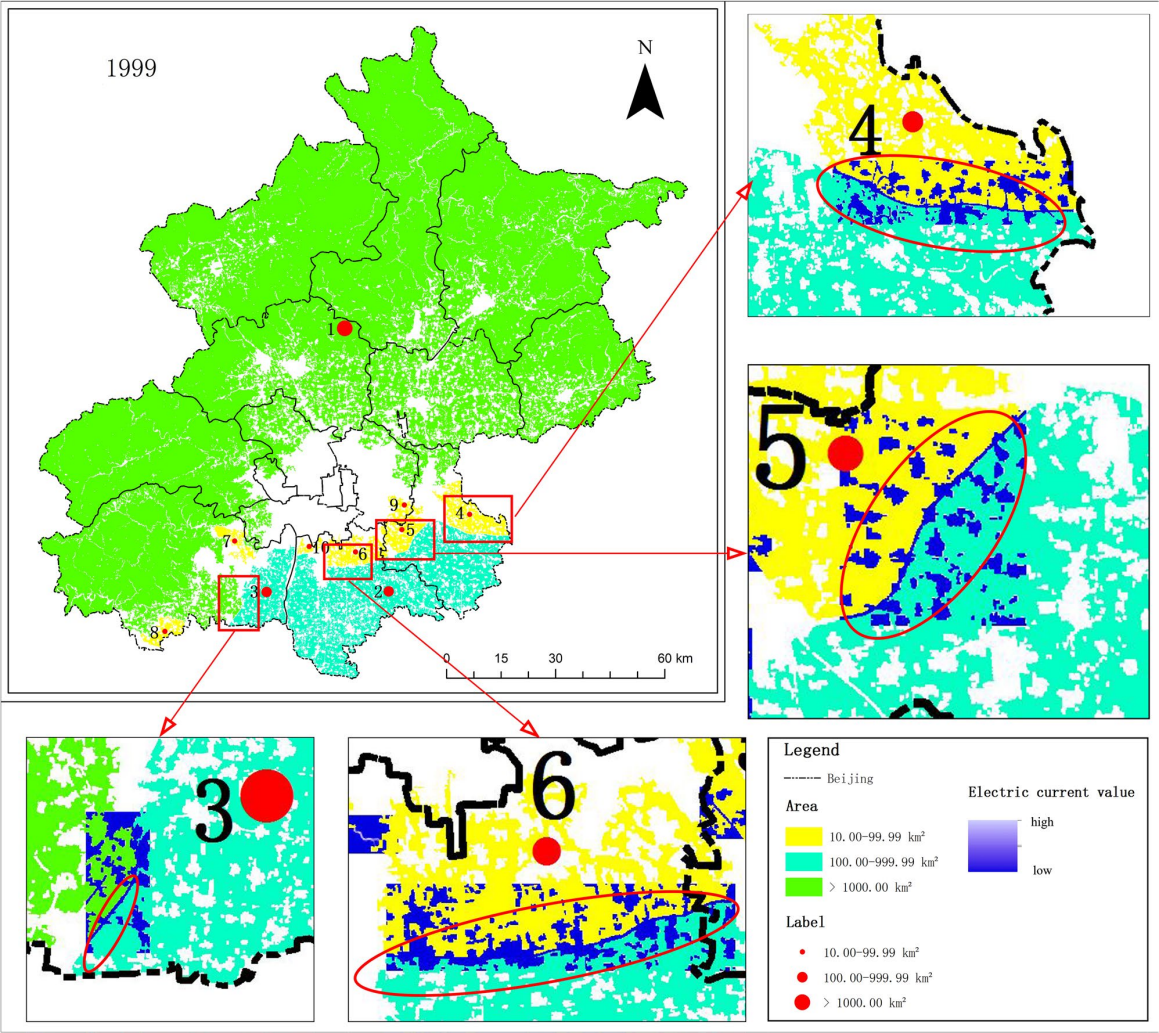
Potential corridors between major cores in 1999. The figure was drawn with ArcGIS sofware version 10.4. (https://www.esri.com/en-us/arcgis/products/arcgis-desktop/overview).

According to the distribution of UGI, Beijing was divided into three regions. Region one was a suburban areas of Huairou, Miyun and Yanqing, where UGI was widely and intensively distributed. The area and distribution pattern of UGI should be maintained in this region. Region two mainly referred to Tongzhou, Daxing and the east of Fangshan. The distribution of UGI in this region was relatively sparse, and the area of a single core was small. Meanwhile, this region was also a key area for urbanization and urban expansion. In the urban planning, we should take the premise of non-destruction of ecological environment and firmly curb the occupation of UGI. Region three mainly referred to the main urban area within the sixth ring road of Beijing, which was the most sparsely distributed area of UGI and also the key optimization area. According to the results of the study, the road network was the key factor affecting the connectivity of UGI. Other scholars have also demonstrated that the traffic road network in Beijing affects the connectivity of ecological networks^47^. Taking the data of 1999 as an example, the connectivity between the major cores of the amplification window was affected by the cut-off of the roads, as shown in Fig. 5. The road network severely affected connectivity between key cores, which can be achieved by building ecological corridors on the roads to enhance the connectivity between UGI without affecting the accessibility of the road. Within the sixth ring road in the central area of the study area, as shown in Fig. 1B(e), construction land and road network in the area were relatively dense, with few large core. In these areas, the connectivity of UGI can be enhanced as much as possible by planting street trees.

## Conclusions

We proposed a framework of spatio-temporal evolution characteristics and prediction analysis of UGI based on MSPA and CA-Markov, which has strong universality and migration. The research framework combined prediction and mathematical morphology, which enables to diagnose the historical, current and future evolutionary characteristics of UGI in a comprehensive manner. The results showed that from 1990 to 2019, the total area of UGI and the area of core in Beijing presented "V" shaped change trend. Further study revealed that the turning point of area and the distribution of barycenter both turned in 2009. From 1990 to 2009, the country's development paid more attention to economic progress, which led to a large influx of people into cities and rapid urban expansion, occupying more cultivated land and forest land. With the inclusion of ecological civilization in the " five-sphere integrated plan" national development strategy at the 18th National Congress of the Communist Party of China in 2012, some departments at all levels actively responded by planting trees and demolishing illegal buildings. The area of UGI was gradually increasing, and the connectivity was gradually increasing. Therefore, there was a clear turning point in the UGI of Beijing in 2009. Among the three prediction scenarios for 2030, the area of UGI under the ecological conservation priority scenario is the largest, accounting for 86.35% of the total area. The area of UGI under the economic development priority scenario is the smallest, accounting for 76.85%. The road network was the main factor affecting connectivity of UGI. Ecological corridors may be built on the road network in the suburbs of Beijing, and street trees may be planted in the city center to promote the connectivity of UGI in Beijing.

In conclusion, clarifying the evolutionary characteristics and mastering the change rules of UGI can better serve the planning and development of low-carbon, livable and high-quality cities. In addition, road network is an important factor in research of UGI. Finally, the next step is to carry out analysis on the street scale with the support of higher resolution images.

## Materials and methods

### Study region

**Figure 6:**
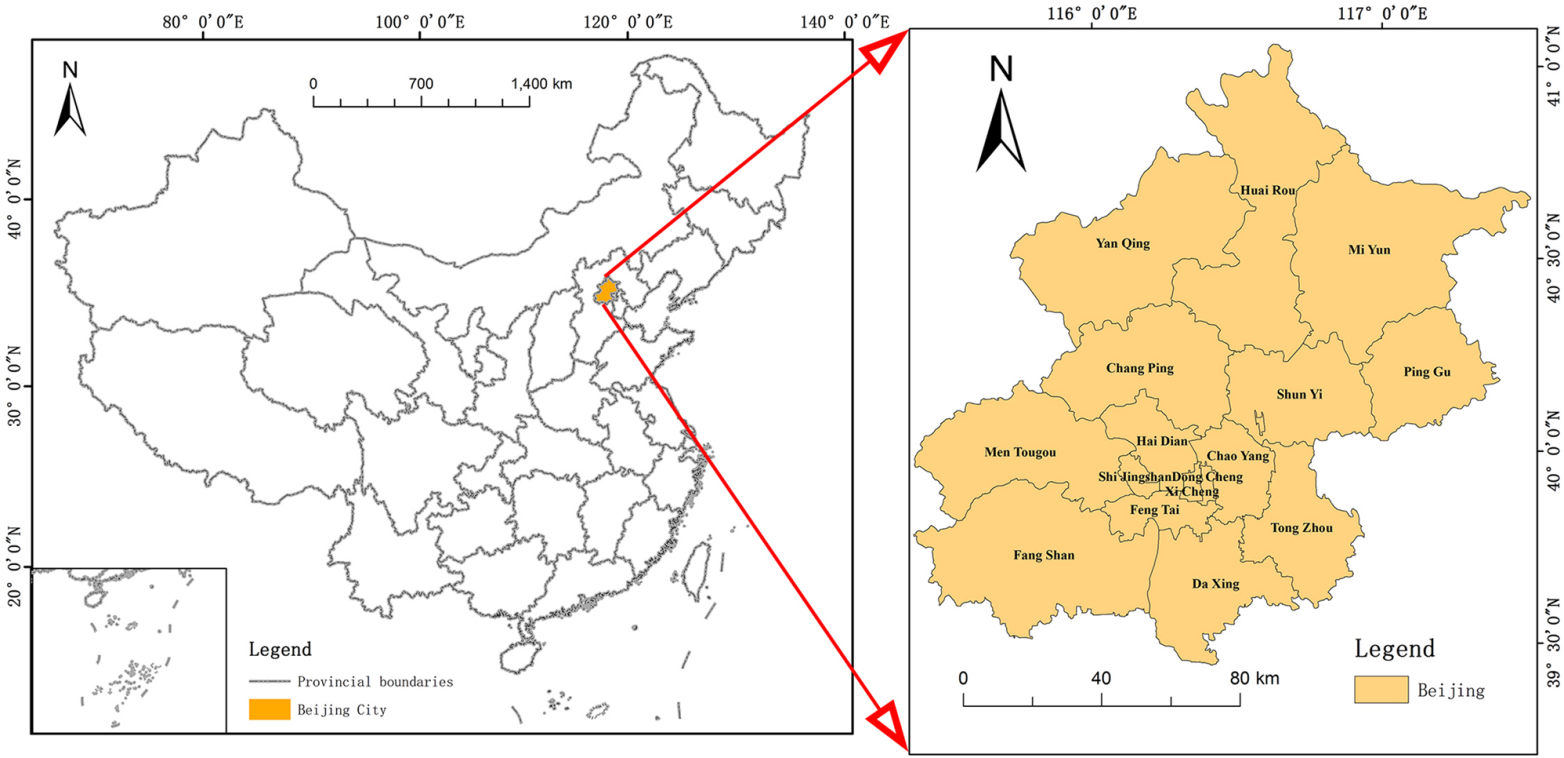
The location of Beijing in China and the administrative zoning map of Beijing. The figure was drawn with ArcGIS sofware version 10.4. (https://www.esri.com/en-us/arcgis/products/arcgis-desktop/overview). The China map data and Beijing boundary data were collected from Resources and Environmental Science and Data Center (http://www.resdc.cn/).

Beijing (39.4°–41.6°N, 115.7°–117.4°E) is located in the central part of China. It is a megacity composed of 16 administrative districts with a total area of 16,410.54 km^2^^43^, as shown in Fig. 6. The terrain of Beijing is high in the northwest and low in the southeast. It is surrounded by mountains in the west, north and northeast. In the southeast, it is a plain. The climate is semi-humid and semi-arid monsoon climate in the warm temperate zone, with high temperature and rainy summer, concentrating 80% of the annual precipitation. The winter is cold and dry, and the spring and autumn are short. The study area is capital of China, a municipality directly under the central government, a national central city, a megacity and a first-tier city in the world. Beijing is also political, cultural, international exchange and technological innovation center of China. In recent years, it has been developing in the direction of livability and high quality. In terms of the size, status and development pattern of the city itself, Beijing has a strong representation, so it is selected as the study area.

### Data sources

Due to the constraints of imaging time, quality, accessibility and continuity of image data, the Landsat remote sensing image data in 1990, 1999, 2009 and 2019 can meet the needs of this study. Image data was downloaded from the Geospatial Data Cloud (http://www.gscloud.cn/home). Details of the data were shown in Table 3, in which the cloud cover in the image was not within the scope of the study area. According to the climate and phenology information of Beijing, we selected the image data of August and September for the study.

**Table 3:** Landsat image data information.

Imaging time	Satellite	Sensor	Track	Resolution	Cloud cover	Overall classification accuracy (%)	Kappa coefficient
1990.9.18	Landsat 5	TM	123.032	30	0.00	96.63	0.931
1990.9.18	Landsat 5	TM	123.033	30	0.00		
1999.8.10	Landsat 5	TM	123.032	30	0.00	94.60	0.909
1999.8.10	Landsat 5	TM	123.033	30	0.00		
2009.9.22	Landsat 5	TM	123.032	30	0.00	93.99	0.901
2009.9.22	Landsat 5	TM	123.033	30	0.00		
2019.9.02	Landsat 8	OLI	123.032	30	0.74	96.58	0.906
2019.9.02	Landsat 8	OLI	123.033	30	0.02		
2019.8.24	Landsat 8	OLI	124.032	30	2.08		

With the help of ENVI 5.3, the land use and cover data of Beijing from 1990 to 2019 were extracted. Firstly, the image data were preprocessed by radiometric correction, atmospheric correction and clipping. The Normalized Difference Vegetation Index (NDVI), Normalized Differences Built-up Index (NDBI) and Modified Normalized Differences Water Index (MNDWI) indices of the images were extracted to prepare for subsequent image classification. Then, the sample points were selected by combining Google high-resolution images, extracted index, road network, POI and other related data. Among them, 70% of the selected sample points were used as classification sample points, and the remaining 30% were used as verification sample points. Finally, the images are classified using support vector machines (SVM), which can find the optimal classification surface among different samples from small samples^48, 49^ According to GB/T 21010-2017, the study area was divided into six categories: cultivated land, forest land, grassland, construction land, water area and unused land. It was validated that the overall classification accuracy was above 93% for all periods, and the kappa coefficients were all greater than 0.90, as shown in Table 3.

### Research framework

**Figure 7:**
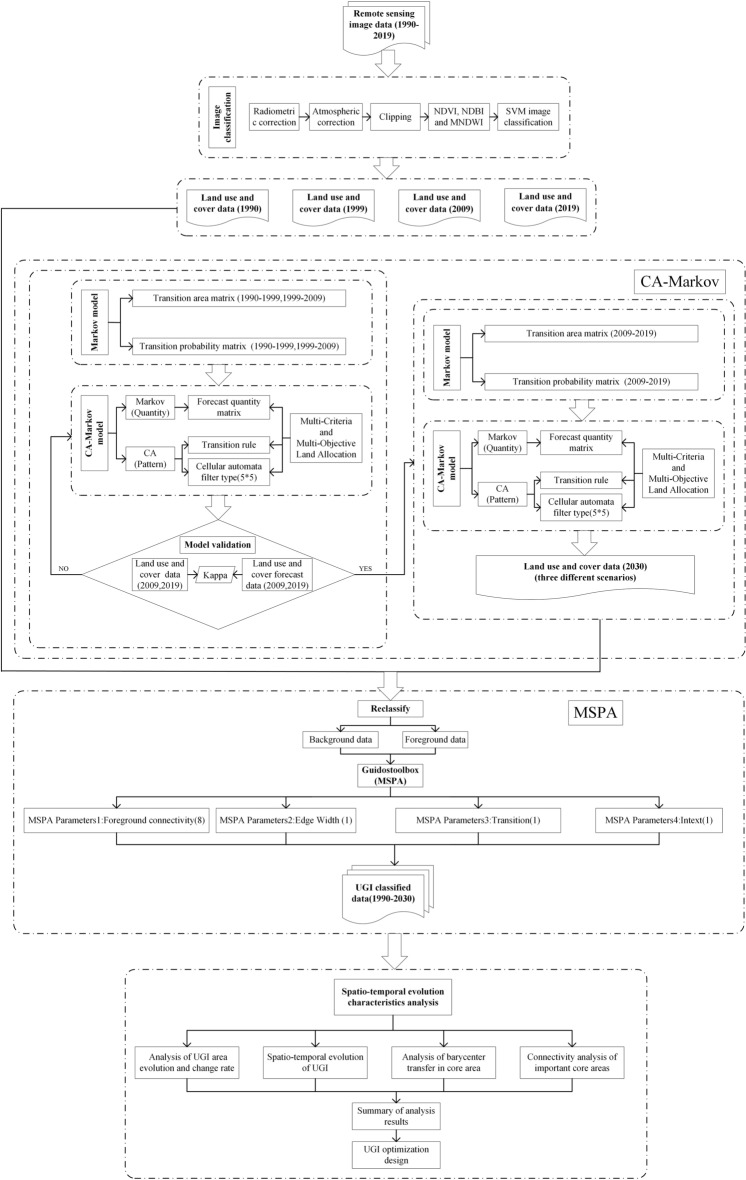
The framework of spatio-temporal evolution characteristics and prediction analysis of UGI.

The framework constructed in this paper realizes the spatio-temporal evolution characteristics and prediction analysis of UGI, as shown in Fig. 7. We used SVM to extract land use and cover data of the study area from 1990 to 2019. Then, the research framework was divided into three modules. The first module was supported by CA–Markov model, and the future land use and cover data were simulated. The history, current and future periods of the study area were linked together to achieve a long time series in tandem. The first module was mainly divided into two parts. One was to verify the feasibility of CA–Markov model by using the existing data. The other was to predict the land use and cover data under three different scenarios in 2030. The second module was to reclassify the land use classification data into foreground and background data, and input them into the Guidooolbox to complete the seven categories of UGI classification. Finally, the spatio-temporal evolution characteristics of UGI were explored by using the analysis methods of change rate index, connectivity and barycenter migration, so as to reasonably optimize UGI and achieve sustainable and high-quality urban development.

### Research method

#### CA–Markov model

In the late 1940s, Stanislaw Ulan and John Von Neumann proposed cellular automata (CA), which is a kind of dynamic model of networks with discrete state, time and space, and strong dynamic. The behavior of CA models is affected by uncertainties arising from the interaction between model elements, structures, and the quality of data sources used as the model input^50, 51^. The model predicts the state of the cell in the next time period by transformation rules based on the current state of each cell in the cell space^52^. Due to its high applicability, the model is widely used in land use change prediction. In the prediction process, both natural and human factors are taken into account to make the prediction results more accurate^51, 53^. In addition, Markov model proposed by Andrey Markov is another commonly used dynamic prediction model in academic research. The model predicts the situation at a future moment by studying the probability of occurrence and change pattern of random events^54^. The CA model is able to express the prediction data and spatial variation trends, but it cannot quantitatively reflect the influence of neighborhood on central cell. However, Markov model has a greater advantage in quantitative prediction, but it cannot be expressed in space^55^. In order to better express the prediction results in time and space, the CA–Markov model was used to predict the land use and cover data of Beijing in 2030 to analyze the change trend of UGI^56^. The kappa coefficient is chosen for the study to evaluate the accuracy of the model. It is a validation method to determine the accuracy of the model, and the coefficient is a common indicator to measure the accuracy of the prediction results^57, 58^. The kappa coefficient is able to evaluate and analyze the prediction results in terms of both quantity and location. The closer its value is to 1, the more perfect it is^58^. When evaluating the prediction accuracy of the model, it is generally considered that the prediction accuracy is high when kappa coefficient is in the range of 0.60–0.80. The prediction accuracy is considered to be very high when the coefficient is in the range of 0.80–1.00^58–60^.

#### MSPA

MSPA is a method based on mathematical morphology for identifying the categories of UGI^61^. According to the survey of the study area, UGI were divided into seven types, namely core, island, edge, perforation, bridge, loop and branch^19^, as shown in Table 4. The tool that we used in our research was Guidostoolbox 3.0 software, which was designed and updated by the Joint Research Center of the European Commission^17^. Researchers have different views on the correspondence between land use types and UGI when conducting studies in different study areas. Beijing is a mega-city with a large demand for green space. Green parks, waters, grasslands, woods and crops in cities all play a positive role in safeguarding urban green space and urban environment. With reference to scholars' research and according to the standard of China's Land Use Status Classification (GB/T 21010-2017), four primary classification types of forest land, cultivated land, grass land and water area were studied as the main scope of UGI in this study^62, 63^. In detail, the construction land and unused land in the land use and cover data were reclassified as background data, setting the image element value to 1. The other categories are reclassified as foreground data, setting the image element value to 2. The data was converted from the original land use data to a TIFF file with a raster size of 30 m × 30 m. Then, the TIFF files were imported into Guidostoolbox 3.0 software for 8-neighborhood MSPA analysis. For the other parameters, the edge width parameter is set to 1, the transition parameter is set to 1, and the intext parameter is set to 1.

**Table 4:** Categories and ecological meanings of UGI^61^.

Categories	Ecological meanings
Core	Habitat patches with large foreground pixel area are the center of green infrastructure network. As the "source" of ecological network, they provide habitat for species and are of great significance to the protection of biodiversity.
Island	Small, isolated and poorly connected green patches are equivalent to "ecological island hopping" in the ecological network. They play a role of media in the ecological network.
Edge	The transition zone between the core and peripheral non green landscape patches can effectively reduce the impact of external landscape man-made interference. They have edge effect.
Perforation	The function of the transition zone between the core and the inner non green landscape patches is similar to that of the edge. They play the role of protecting the core and have the edge effect.
Bridge	The narrow areas with high connectivity between two adjacent cores represent corridors connecting patches in the ecological network. They are important for biological migration, energy exchange and landscape connectivity.
Loop	The inner corridor connecting the same core is the channel for species diffusion and energy exchange within the core patch.
Branch	They are areas where only one end is connected to an edge, bridge, ring, or perforation.

#### Rate of change index of UGI

In order to quantitatively study the range and rate of change of UGI’s area, we proposed single change rate index and comprehensive change rate index of UGI based to the concepts of single land use dynamic degree and comprehensive land use dynamic degree^64^, and its expressions are as follows:

1 $${P}_{s}=\frac{{U}_{b}-{U}_{a}}{{U}_{a}}\times \frac{1}{T}\times 100\%$$

where ${P}_{s}$ is the single change rate index. ${U}_{a}$ and ${U}_{b}$ represent the area of the same categories of UGI at the beginning and end of the study period, with the unit of km^2^. $T$ represents the beginning and end of the study period, with the unit of year.

2 $${P}_{c}=\frac{(\sum_{i=1}^{n}{\Delta U}_{i-j})}{(2\sum_{i=1}^{n}{U}_{i})}\times \frac{1}{T}\times 100\%$$

where, ${P}_{c}$ is the comprehensive change rate index. ${U}_{i}$ represents the area of a certain category($i$) of UGI at the beginning of the study, and ${\Delta U}_{i-j}$ represents the absolute value of the conversion of category ($i$) to non-category($i$) of UGI during the study period, with the unit of km^2^. $T$ represents the beginning and end of the study period, with the unit of year.

#### Connectivity analysis

We analyzed the potential corridors and pinch points of UGI with the help of linkage paths tool and pinch point mapper tool. The linkage paths tool was used to identify the potential corridors with the help of core vector files and regional resistance grids. The resistance grids used in this study were drawn from the land use and cover maps and categories maps of UGI, and each grid value represented the energy consumption, difficulty or death risk value of wild animals through the grid ^65^. In addition, the use of pinch point mapper tool required the auxiliary support of linkage mapper toolbox. This tool used the Ohm's law principle to calculate the pinch point, indicating that some energy was required to pass through two unconnected UGI regions. In detail, the raster with small resistance consumed less energy, which facilitated the connection and construction of ecological corridor. On the contrary, the raster with large resistance consumed more energy, and it was more difficult to connect and construct the ecological corridor.

#### Barycenter migration

The overall characteristics of spatial variation of UGI can be expressed intuitively by barycenter migration. By analyzing the migration direction of barycenter of UGI, the distribution characteristics of spatial variation of UGI can be understood to a certain extent. The calculation formula of barycenter coordinate is as follows^66^:

3 $${X}_{t}=\frac{\sum_{i=1}^{n}({C}_{ti}\times {X}_{i})}{\sum_{i=1}^{n}{C}_{ti}}$$

4 $${Y}_{t}=\frac{\sum_{i=1}^{n}({C}_{ti}\times {Y}_{i})}{\sum_{i=1}^{n}{C}_{ti}}$$

where ${X}_{t}$ and ${Y}_{t}$ respectively represent the longitude and latitude coordinates of the barycenter of a certain category of UGI in the year($t$).${C}_{ti}$ represents the area of the patch($i$) of a certain category of UGI in the year($t$). ${X}_{i}$ and ${Y}_{i}$ represent the longitude and latitude coordinates of the geometric center of the patch($i$) of a certain category of UGI respectively. Taking the core shape file from 1990 as an example, the X and Y coordinates of the geometric center of each core were calculated separately with the assistance of ArcGIS 10.4. Calculation of the coordinates of the barycenter was achieved based on the above formulas and the area of the core. Then, the coordinates of the barycenter of each year's core were calculated in turn, and the migration pattern was explored.

## Data Availability

The China map data and Beijing boundary data can be collected from Resources and Environmental Science and Data Center (http://www.resdc.cn/). The Landsat remote sensing image data in 1990, 1999, 2009 and 2019 can be downloaded from the Geospatial Data Cloud (http://www.gscloud.cn/home). Due to the requirements of the author's dissertation and the needs of further experiments, the GI datasets of Beijing obtained through processing in this study are not convenient for direct public access. But are available from the corresponding author on reasonable request (email: zhengxq@cugb.edu.cn; zxqsd@126.com).
